# Mechanisms of Perinatal Brain Injury

**DOI:** 10.1155/2012/157858

**Published:** 2012-07-11

**Authors:** Robin L. Haynes, Tara M. DeSilva, Jianrong Li

**Affiliations:** ^1^Department of Pathology, Boston Children's Hospital, Harvard Medical School, Boston, MA 02115, USA; ^2^Departments of Physical Medicine and Rehabilitation and Neurobiology, Center for Glial Biology in Medicine, University of Alabama at Birmingham, Birmingham, AL 35294, USA; ^3^Department of Veterinary Integrative Biosciences, Texas A&M University, College Station, TX 77843, USA

The guest editors of this journal are pleased to introduce this special issue on the mechanisms of perinatal brain injury. The purpose of this issue is to highlight recent developments toward our understanding of both grey and white matter injury in the perinatal brain. Over the last decade, there has been significant advancement in our understanding of the mechanisms underlying this injury. The progression of the field stems from many factors including: (1) the development and refinement of experimental models; (2) advances in modern imaging technologies, such as magnetic resonance imaging and diffusion tensor imaging; (3) detailed characterization of the injury, both in human and animals, on the anatomical, cellular, and subcellular levels and (4) identification of the developmental factors that underlie the vulnerability of the perinatal brain to such injury. Here we present nine review and two research articles that together highlight these advancements, as summarized below. It has been our honor to work with each of the contributing scientists, and we thank them for their commitment to this Special Issue. 

The review by C. Thornton et al. summarizes our current understanding of the “*Molecular mechanisms of perinatal brain injury,*” with a focus on mitochondrial functional impairment and apoptotic events during the secondary injury phase. The authors also present the most up-to-date intervention strategies targeting the different stages of brain injury.

V. Ten and A. Starkov further explore the role of mitochondria in hypoxic-ischemic (H-I) injury and provide evidence for the mitochondrial production of reactive oxygen species as a pathogenic mechanism underlying this injury. The authors summarize experimental data delineating mitochondrial sources of reactive oxygen species and their specific targets in neonatal animals.

Necrotic cell death is well documented in H-I brain injury and is traditionally thought uncontrollable. In another review, R. Chavez-Valdez et al. introduce the recently identified RIP1/3 kinase dependent programmed necrosis pathway to the field of neonatal brain injury and propose an apoptosis-necrosis cell death “continuum” for cellular degeneration. The authors highlight experimental evidence supporting a prominent role of programmed necrosis in neonatal H-I brain injury. 

Another paper by C. Hill and R. Fitch explores the underlying basis of the increased vulnerability of male infants to H-I injury and their increased incidence of cognitive deficits associated with that injury. The authors discuss hormonal factors underlying these differences as well as sex-specific differences in H-I-induced cell death pathways.

In the review by K. Buller et al., the effects of perinatal H-I injury on the serotonergic system are summarized. The potential use of anti-inflammatory interventions to alleviate this injury is also discussed. Disruption of this neurotransmitter system may underlie the cardiorespiratory, cognitive, and attention deficits observed in survivors of prematurity.

Changing the focus from H-I injury to inflammation and infection, C. Mallard and X. Wang summarize clinical and experimental evidence for neonatal sepsis and increased vulnerability of the immature brain and discuss the involvement of Toll-like receptors (TLRs) in perinatal brain injury.

Continuing in the area of inflammation and infection, R. McAdams and S. Juul address the initiation and activation of cytokines and inflammatory cells in the perinatal brain and their detrimental short and long-term consequences. They highlight the importance of understanding the dynamic structure of the blood brain barrier, as well as the relatively unknown mechanisms that impair its integrity allowing immune cells direct access to the brain. 

In the first of two research articles, D. Selip et al. characterize, in a rodent model of H-I, the spectrum of grey matter damage in association with the white matter damage once thought to be dominant in H-I injury. The authors characterize the regional susceptibility to H-I injury in terms of the inflammatory response, white matter injury and myelin loss, neuronal degeneration, and axonal injury.

Our second research article by K. Shrivastava et al. provides an extensive characterization of the glia/inflammatory response following H-I using a common model of injury thought to spare the contralateral hemisphere. This study extends our knowledge of this model by characterizing a subtle inflammatory response in the contralateral brain, and provides important information regarding the timing of the insult in relationship to the initiation of inflammatory signaling cascades.

The paper by V. Biran et al. highlights a novel area of the brain—the cerebellum—that has emerged as an important attribute of perinatal brain injury through imaging studies. This review provides an extensive detailed analysis of cerebellar development, and the implications of disrupting its normal maturation as a consequence of perinatal brain injury. 

Finally H. Kinney and J. Volpe stress the importance of developing and using animal models with a careful consideration of human perinatal brain development and injury. This paper emphasizes the need to model perinatal brain injury as an encephalopathy of prematurity characterized by a combination of both grey and white matter lesions. 

